# Satellite Navigation Message Authentication in GNSS: Research on Message Scheduler for SBAS L1

**DOI:** 10.3390/s24020360

**Published:** 2024-01-07

**Authors:** Jiangyao Song, Ting Liu, Xiao Chen, Zhongwang Wu

**Affiliations:** 1 Space Engineering University, Beijing 101416, China; s1242620461@126.com; 2 Aerospace Information Research Institute, Chinese Academy of Sciences, Beijing 100094, China; liuting101015@aircas.ac.cn (T.L.); chenxiao@aircas.ac.cn (X.C.)

**Keywords:** SBAS, authentication, TESLA, fixed time sequence dynamic message scheduler

## Abstract

SBAS is mainly used in civil aviation and navigation, and will be applied to autonomous driving in the future. Given the open signal format of the Satellite Based Augmentation System (SBAS), which exposes security threats such as spoofing attacks, the utilization of SBAS navigation message authentication technology can improve the SBAS anti-spoofing ability from the system side. SBAS message authentication technology has become the future direction of SBAS system development. However, during the initial design of SBAS on L1, message authentication technology was not considered, and the addition of authentication messages will lead to further strain on existing message bandwidth resources. Therefore, in response to the issue of insufficient bandwidth resources after adding authentication messages to SBAS L1, a study on message scheduler for SBAS L1 authentication was conducted. A fixed time sequence dynamic message scheduler for incorporating authentication messages was proposed. This scheduler reduces the frequency of broadcasting clock error parameters to mitigate the impact of adding authentication messages. Furthermore, an optimized fixed time sequence dynamic message scheduler based on SBAS clock error messages was introduced. The results show that the message scheduler can not only improve the flexibility of SBAS message broadcasting, but also shorten the update interval of various types of messages under the premise of meeting the maximum update interval requirement. With minimal impact on the maximum message update interval, it improves the integrity, authenticity, and availability of messages. This approach can increase the effective message ratio in SBAS to over 91%, and the optimal solution reduces the initial user positioning time to 26 s.

## 1. Introduction

SBAS, as a wide-area augmentation system, broadcasts differential correction information and integrity data to users. Satellite Based Augmentation System (SBAS) is applied in the field of life safety and meets the navigation requirements of civil aviation aircraft from en-route to precision approach, which puts higher demands on its safety. With the advancement of Dual-Frequency Multi-Constellation (DFMC) technology in SBAS, its services can play an important role in the fields of aviation, navigation, railway, and other high-integrity requirements. However, SBAS currently relies on open signal formats for broadcasting, and the integrity cannot be guaranteed, whose signal has a security risk of spoofing attacks [1]. Therefore, improving the security of SBAS services has become a critical priority of SBAS technology development [2], and message authentication for SBAS offers a viable solution to address this issue.

In response to spoofing attacks, a system-side solution known as SBAS message authentication has been proposed, which has been researched both domestically and internationally. The concept of SBAS message authentication was initially introduced by American scholar Logan Scott in 2003. This concept involves the integration of authentication messages into the existing SBAS messages, allowing users to employ these authentication messages to verify the integrity and authenticity of messages, thereby enhancing anti-spoofing capabilities. In 2016, the European Union proposed the message authentication plan for the European Geostationary Navigation Overlay Service (EGNOS) and developed the EGNOS Authentication Security Test-bed (EAST) project with the aim of achieving EGNOS authentication services and evaluating their performance [3]. Subsequently, both Europe and the United States further proposed SBAS authentication schemes based on the Timed Efficient Streaming Loss-tolerant Authentication Protocol (TESLA) and Digital Signature (DS) [4]. Due to the limited bandwidth of SBAS broadcast signals, the TESLA authentication scheme is gradually becoming the preferred scheme for SBAS message authentication due to its low communication overhead and good resistance to packet loss compared to the Elliptic Curve Digital Signature Algorithm (ECDSA) scheme [5,6]. In 2021, the Federal Aviation Administration (FAA) in the United States proposed authentication planning on the L1 frequency [7,8]. In 2022, Stanford University in the United States preliminarily designed SBAS message formats and defined new message types for broadcasting authentication messages. Although research on message authentication has been ongoing for many years, it has not yet been studied together with the message scheduler in the field of L1 frequency. Combining these two aspects presents a challenge: the bandwidth resources of SBAS L1 frequency are already relatively limited, and the addition of authentication messages will have an impact on the existing message scheduler.

In terms of the message scheduler, the existing SBAS message schedulers primarily include the fixed sequence message scheduler, dynamic sequence message scheduler and fixed time sequence dynamic message scheduler. In the fixed time sequence dynamic message scheduler, some messages are broadcast at fixed intervals, while the remaining messages are broadcast dynamically. Compared to the fixed sequence message scheduler proposed by Stanford, this approach does not necessitate strict adherence to predefined sequences, which can improve the flexibility of SBAS message broadcasting. It reduces the broadcast of ineffective messages, enhances the efficiency of data channel broadcasting, increases the proportion of effective messages, and shortens the update interval of various SBAS messages [9]. Moreover, it avoids the occurrence of multiple message timeouts at the same time, as observed in the dynamic sequence message scheduler introduced by Yun Y. and colleagues in South Korea [9]. In 2021, Zhang Jingcan et al. from China proposed a fixed time sequence dynamic message scheduler for broadcasting the Beidou Satellite-Based Augmentation System (BDSBAS) messages. In comparison to the current fixed-style broadcasting of BDSBAS messages, this method enhances the flexibility of message broadcasting, shortens various message update intervals, reduces the user’s initial positioning time by approximately 11.7%, and better ensures the integrity and continuity of BDSBAS services [9]. All of the aforementioned strategies were developed without considering authentication messages. Authentication messages for SBAS will occupy at least 1/6 of the message bandwidth. If the authentication messages are directly broadcast according to the above two schedulers, it will have a negative impact on the broadcasting of the original messages [10], which may lead to timeouts in some message broadcasting. Therefore, it is necessary to re-design the message scheduler.

This paper addresses the issue of insufficient bandwidth after adding authentication messages and conducts research on message scheduler for L1 SBAS authentication. In the field of navigation, clock error is not an obligatory condition, and therefore, the optimization of clock error can be considered. There are two approaches to decreasing clock error: reducing the frequency and reducing the number of satellites. The existing satellite clock error is 6 s, and there is consideration of decreasing it to 60 s. At the same time, in order to ensure a strong constraint that 6 s is the time for an SBAS to detect the service integrity [8], new integrity messages need to be added. Another approach is to reduce the number of satellites that need to be broadcast, as the current number of broadcastable satellites is often greater than the actual number used. This approach helps conserve bandwidth. In this study, four different schedulers were designed and compared to determine the optimal message scheduler including authentication. With the selected scheme, the proportion of effective SBAS messages can reach over 96%, and the user’s time to first fix time (TTFF) is reduced to 26 s. The paper begins by providing a brief explanation of the SBAS message authentication principles, then proposes some message schedulers based on the TESLA protocol with added authentication. Finally, simulations and analyses are carried out for the method to verify the feasibility of adding authentication messages MT20 and MT21, evaluate the performance of four different types of fixed time sequence dynamic message schedulers after adding authentication messages, and select the best optimization scheduler.

## 2. Basic Principles of SBAS Message Authentication

### 2.1. The Principles of SBAS Message Authentication

SBAS message authentication relies on cryptographic algorithms to incorporate authentication tags into satellite broadcast messages. Once users receive these tags, they can verify whether the navigation messages have been tampered with or counterfeited by attackers [11]. The main objective of SBAS message authentication is to ensure the integrity of SBAS messages. Without affecting the user’s normal access to the basic SBAS service, it provides more secure and credible navigation services to the users by increasing the verification of the message integrity and the source of the signal to improve the SBAS message anti-spoofing attack capability [12].

**Figure 1:**
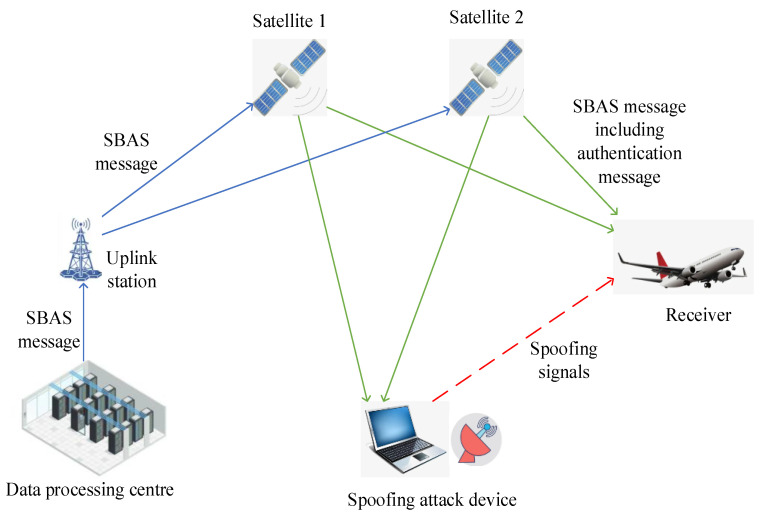
SBAS system components.

After the addition of authentication, the main components of SBAS are shown in Figure 1. The data processing center generates the authentication messages according to the cryptographic algorithm and packages them alongside SBAS messages. The uplink station modulates the uploading signal according to the packaged message and sends it to the Geostationary Orbit (GEO) satellite. The GEO satellites relay the uplink station’s signals, forming SBAS broadcast signals. Receivers utilize the received SBAS signals containing authentication messages to conduct source identity authentication, while the spoofing attacking device finds it challenging to forge authentication messages. Receivers can distinguish whether the SBAS message originates from the real GEO satellite and whether it has been tampered with. This significantly enhances resistance to SBAS spoofing attacks [7] and meets the requirements for anti-spoofing protection.

### 2.2. Message Authentication Protocol

The SBAS message authentication protocol employs the TESLA protocol, which is a relatively secure source authentication protocol applied to multicast and broadcast data streams. It constructs an asymmetric system through the delayed dissemination of keys to ensure the security of the protocol. The TESLA protocol offers low communication overhead, robust resistance to packet loss, and resistance to quantum computing attacks. It is well-suited for use in broadcast authentication scenarios.

#### 2.2.1. The Components of the TESLA Protocol

**Figure 2:**
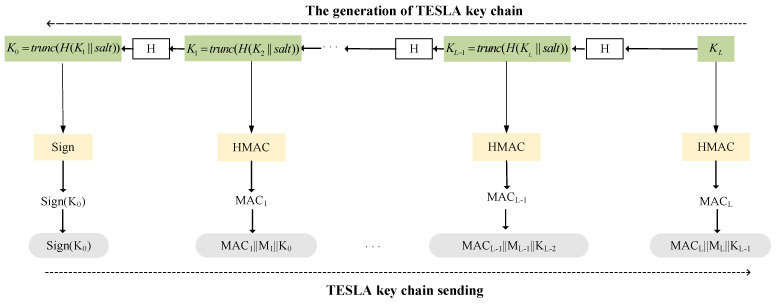
TESLA key chain.

In the TESLA protocol, identity authentication is provided by generating a Message Authentication Code (MAC) [10]. This process primarily consists of one-way key chain generation, generation of *MAC*, and delayed disclosure of keys. The procedure is illustrated in Figure 2, where *K*_0_ represents the root key, *K_i_* represents the keys, *H*() denotes a one-way hash function, Sign stands for the digital signature, *M_i_* is the navigation message, and MAC is the message authentication code.

The generation of the key chain begins by defining its length, denoted as *L*. The key chain starts with a randomly generated encryption key *K_L_*. The key chain is created using a one-way hash function [13], which ensures that once a key *K_i_* is obtained, it cannot be used to deduce future keys *K_i+j_*, where *∀j* > 0. The generated hash values serve as keys [14]. To increase the randomness in this process, a “salt” is introduced. The key is transmitted in the form of a message. Due to bandwidth limitations, the length of the key must not be excessively long and needs to be truncated to an acceptable length using a truncation function. Moreover, to prevent attackers from tampering with or forging the entire key chain, the sender must digitally sign the root key *K_0_*, and send the digital signature to the users.

(1) $$K_{i}=trunc(H(K_{i+1}||salt))$$

The generation of message authentication code, in order to confirm the integrity of the message, generates the *MAC* from the key and the message based on the function *HMAC*() of the encryption algorithm [15]. In this process, the key chain used to generate the MAC operates in the reverse order of the key chain generation. It starts with the root key *K*_0_ and ends with *K_L_*.

(2) $$MAC_{L}=HMAC(K_{L},M_{L})$$

The delayed disclosure of keys process means that each key is delayed to be disclosed to the user. After broadcasting the MAC, the sender delays δ time to send the key used to generate this MAC, so that the user can only use this delayed key to authenticate previous messages.

#### 2.2.2. Key Management Scheme

Key management primarily involves the generation, storage, distribution, and updating of key pairs [16]. The TESLA protocol employs a three-tier key management scheme to ensure the security of the keys. The three tiers of keys are as follows:

(1) Level 1 key (L1) is the CA (Certificate Authority) key pair issued by the certificate management organization.
(2) Level 2 key (L2) is the system public-private key pair used for authenticating the TESLA key chain root key.
(3) Level 3 key (L3) is the key used for message authentication in the TESLA key chain.

**Figure 3:**
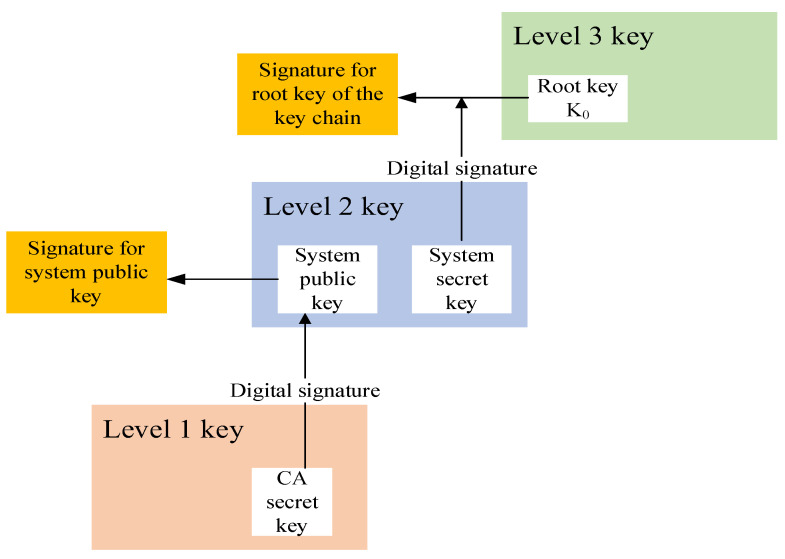
Schematic of TESLA three-tier key management scheme.

As shown in Figure 3, the keys within the TESLA key chain are primarily used for authenticating SBAS messages, in which the root key can be used to ensure the security of the key chain [17]. The root key of the key chain is signed with the L2 system private key, and the authenticity of the root key of the key chain is verified with the L2 system public key. The L2 system public key is signed using the CA private key through the ECDSA algorithm, and the authenticity of the received L2 system public key is verified with the CA public key.

### 2.3. Design of Authentication Messages

**Figure 4:**
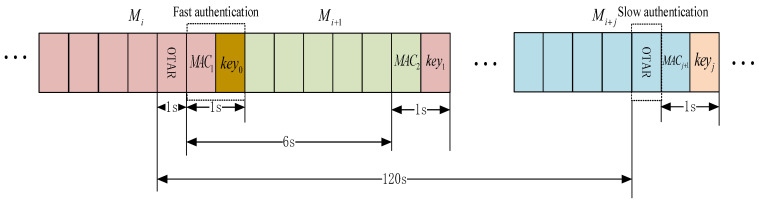
Intertwine authentication messages with other SBAS messages.

According to the TESLA protocol and the key management scheme, authentication messages are primarily divided into two categories:

One category belongs to the TESLA protocol. These are fast authentication messages that include a MAC and a delayed key. This category of messages can satisfy the 6-s update interval and achieve the authentication of the integrity. Figure 4 shows the broadcast structure of the SBAS authentication message.

The first five messages, along with MAC_s,_ are broadcast initially, and the keys used to generate MAC_s_ will be broadcast subsequently. From the color coding in Figure 4, it can be observed that the red Key_1_ is associated with the previous set of MAC_s_ rather than the MAC_s_ contained within the same message. For every 6-s message frame, 5 s are used to broadcast SBAS differential and integrity messages, and 1 s is dedicated to broadcasting SBAS authentication messages. The MAC and Key are transmitted together, occupying 1 s, and the keys will be broadcast to everyone with a delay of 6 s.

The other category belongs to the TESLA three-level key management system, which is a slow authentication message containing the root key of the TESLA key chain, the second-level system public key and signature, and the CA public key. Since the key management information is broadcast over the air, this type of message can be called Over the Air Rekeying (OTAR) message. Due to the slower update frequency of key management information, the update interval for this type of message is longer.

### 2.4. Single-Frequency SBAS Message Format

In view of the new message contents required for SBAS message authentication, SBAS Standards and Recommended Practices (SARPs) have added MT20 and MT21 (originally reserved MT). MT20 is used for transmitting authentication messages with a maximum update interval of 6 s. MT21 is used for transmitting OTAR messages, which include three types: one is the Root key of the TESLA key chain and corresponding signature with a maximum update interval of 120 s; another is the Level 2 public key and corresponding signature with a maximum update interval of 360 s, and the other is the CA public key with a maximum update interval of 360 s. The newly defined data types, message types, and maximum update intervals for authentication messages are presented in Table 1.

The various types of messages in SBAS messages contain different information and have varying degrees of timeliness. In general, users prefer to receive data such as satellite ephemeris and satellite clock corrections as quickly as possible to aid in rapid positioning. Integrity information, such as system operating status, should also be provided promptly. The urgency for receiving other types of data may be somewhat lower. In terms of the overall structure, navigation messages should be able to meet the broadcast requirements for data with different levels of timeliness [18]. Single-frequency SBAS supports a total of 64 message types, with each message consisting of 250 bits and being broadcast within 1 s [19]. Table 2 below lists the main message types and their maximum update intervals for SBAS L1. MT1 provides a Pseudo Random Noise (PRN), which specifies the PRN number of the target satellite for the augmentation message. MT2–5 and MT24 provide fast corrections, MT6 offers integrity monitoring, and MT24 provides long-term corrections. MT20 broadcasts SBAS L1 identity authentication information, while MT21 broadcasts OTAR messages.

## 3. Fixed Time Sequence Dynamic Message Scheduler Based on SBAS Clock Error Message Optimization

### 3.1. Problems with Existing SBAS Message Scheduler

At the current stage, the SBAS message schedulers primarily include the fixed sequence message scheduler, dynamic sequence message scheduler and fixed time sequence dynamic message scheduler. Take a fixed sequence message scheduler as an example.

**Figure 5:**
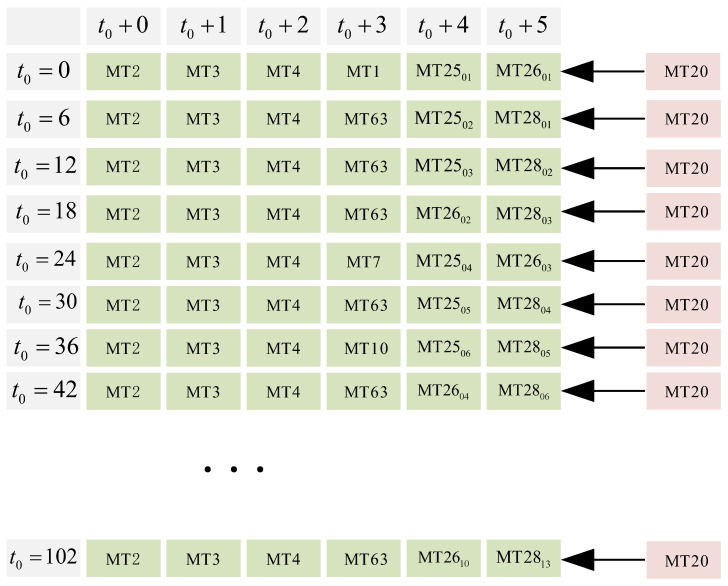
Schematic of Stanford’s fixed sequence message scheduler for joining authentication messages.

According to Figure 5, it can be observed that directly replacing the last message every 6 s with the authentication message MT20 will have a significant impact on the existing messages. This may cause some message types to not adhere to their maximum message update intervals. Therefore, there is a need to design a message scheduler that incorporates authentication messages while still meeting the maximum update intervals and efficiently utilizing available bandwidth as much as possible.

### 3.2. Design Ideas

**Figure 6:**
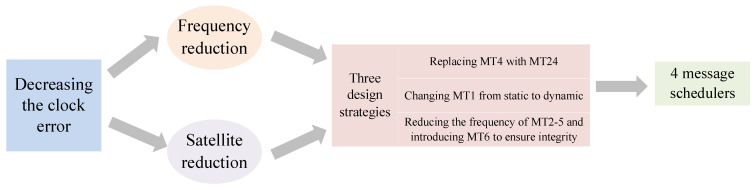
Design idea.

For SBAS L1 message authentication, with the goal of optimizing bandwidth while ensuring the broadcast frequency of the message, it is necessary to optimize the scheduler of the existing message type to reduce the impact on the original SBAS messages. The design idea is shown in Figure 6.

Currently, the actual broadcast interval for clock error is 6 s, and bandwidth is considered to be saved by decreasing the clock error to provide support for authentication message joining. There are two main approaches to decreasing the clock error: frequency reduction and satellite reduction. Frequency reduction is to reduce the broadcast frequency of the message, and satellite reduction is to decrease the number of broadcastable satellites. Three design strategies can be employed to achieve frequency and satellite reduction:

- Replacing MT4 with MT24
- Changing MT1 from static to dynamic
- Reducing the frequency of MT2–5 and introducing MT6 to ensure integrity.

Further, four specific fixed time sequence dynamic message schedulers are designed based on these three designs, which are described in detail below.

### 3.3. SBAS Decreased Clock Error Message Design

In accordance with integrity requirements, MT20 needs to be updated every 6 s and occupies 1/6 of the message bandwidth. Therefore, the addition of authentication messages will impact the existing messages. It’s necessary to optimize the scheduler of SBAS messages to ensure that the inclusion of authentication services does not degrade the performance of the original SBAS services. Three design strategies are put forth to optimize the scheduler of SBAS messages by reducing the clock error frequency for MT2–5.

(1) Replacing MT4 with MT24

**Figure 7:**
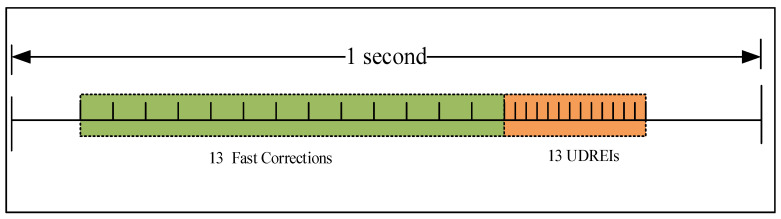
MT2–5 fast correction message format.

**Figure 8:**
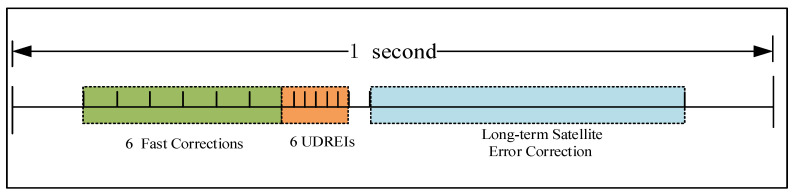
MT24 mixed corrections message format.

MT2–5 messages include fast correction and integrity information, as shown in Figure 7, with integrity parameters including User Differential Range Error (UDRE). Each type can broadcast fast correction and integrity information for 13 satellites. MT2 broadcasts data corresponding to the first 13 satellites, MT3 broadcasts data corresponding to satellites 14–26, and MT4 broadcasts data corresponding to satellites 27–39. As shown in Figure 8, MT24 broadcasts a mix of satellite fast corrections and long-term corrections, with the first half of which consists of 6 fast datasets (where the UDREI is 4 bits). If there are six or fewer satellites, they may be included in the hybrid correction information of MT24, making it feasible to replace MT4 with MT24. The first proposed scheduler utilizes MT24 instead of MT4, and static MT1, MT24, MT28, and fast correction messages are in a fixed time sequence.

(2) Dynamic MT1 to reduce MT4

MT1 is responsible for broadcasting satellite masks, which consist of 210 ordered slots. Each slot indicates whether the corresponding satellite, as defined in Table 3, is providing data. The PRN mask can only be 0 or 1, where “0” represents that the satellite is not observed, and “1” represents that the satellite message can be received.

The current SBAS system uses static MT1, where the PRN mask is always set to “1”, indicating that all satellites are monitored by default. However, in terms of integrity, if a satellite is not observed in reality, the UDREI for that satellite will be set to “14”. Static MT1 is always constant, but in practice, it is not necessary to broadcast such a large number of satellites. Broadcasting all MT2–4 messages can result in significant bandwidth usage.

The appearance of dynamic MT1 indicates the occurrence of satellite PRN number change, i.e., the replacement of other satellites. When there are not many satellites, the majority of fast correction messages are transmitted through MT2 and MT3. Therefore, the number of MT4 can be reduced accordingly. The second proposed scheduler adopts this approach, where the fixed time sequence MT1 is no longer static but dynamic. The messages do not include MT24. MT28 and fast correction messages follow a fixed time sequence.

(3) Reducing the frequency of MT2–5

Considering that SBAS clock error fast correction parameters are primarily used to mitigate GPS Selective Availability (SA), which was discontinued by GPS in 2000, it is contemplated to reduce the frequency of MT2–5 transmissions. This reduction specifically pertains to clock error. However, as shown in Figure 7, MT2–5 also contain integrity information. Therefore, it’s necessary to consider using other integrity message types, such as MT24 and MT6, to replace the integrity information within MT2–5. The 6-s integrity characteristic of the messages cannot be altered [22], and the current frequency of MT2–5, which broadcasts every 6 s, is already quite high. Therefore, it can be appropriately reduced to allocate bandwidth for authentication messages. The clock error bias between the reduced frequency and the 6-s should not be too large, and the clock error can be observed through the Pseudo Range Correction (PRC).

Each of the MT2–5 messages contains 13 12-bit PRC. According to the requirements, the resolution of the 12-bit PRC is 0.125 m, with a valid range from −256.000 m to 255.875 m. Users can calculate the current *PRC* PRC(t) based on the *PRC* information $PRC(t_{of})$ broadcast in the message, using the following formula:

(3) $$PRC(t)=PRC(t_{of})+RRC(t_{of})\times (t-t_{of})$$

(4) $$RRC(t_{of})=\frac{PRC_{current}-PRC_{previous}}{\Delta t}$$

(5) $$\Delta t=t_{of}-t_{of,previous}$$

where: [22]

$PRC_{current}$ = latest received PRC (broadcast by MT2–5, 24)

$PRC_{previous}$ = PRC received before $PRC_{current}$ (broadcast by MT2–5, 24)

$t_{of}$ = the reference moment of $PRC_{current}$

$t_{of,previous}$ = the reference moment of $PRC_{previous}$

Since MT5 is only broadcast when 40 or more satellites are specified, MT5 is not considered in the message scheduler of this paper. When reducing the frequency of MT2–5, MT6 is added to meet the 6-s integrity requirement.

### 3.4. Feasibility Analysis of Decreasing Clock Error

(1) Feasibility of replacing MT4

**Figure 9:**
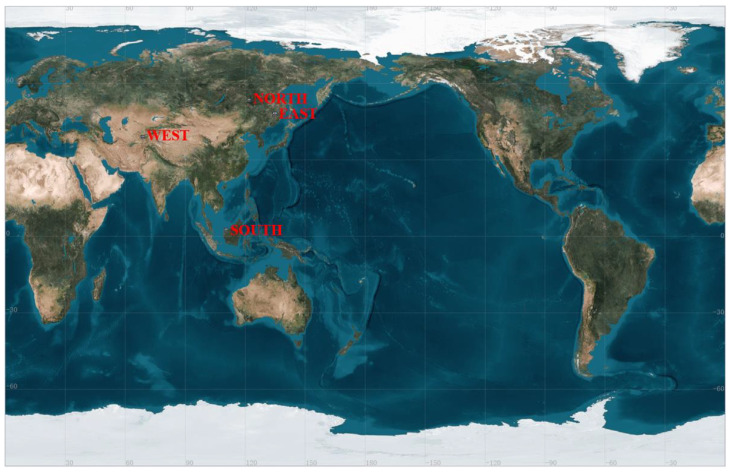
Schematic diagram of the simulation area.

Simulation was conducted for the design of replacing MT4, using broadcast ephemeris provided by the International GNSS Service (IGS) Data Center, and simulating GPS-visible satellites based on simulation software. The simulation considered the coverage area for BDSBAS applications and selected four observation points in different regions of China (Westernmost Point: Pamir Plateau in Xinjiang, 73° E, 39° N; Northernmost Point: Mohe, 123° E, 53° N; Easternmost Point: Intersection of the main channels of Heilongjiang and Wusuli River, 135° E, 48° N; Southernmost Point: Zengmu Ansha, 112° E, 3° N), as shown in Figure 9. The period of GPS satellites orbiting the Earth is approximately 12 h, which means they orbit the Earth twice within a 24-h day. Therefore, one day’s worth of data can represent changes in the number of GPS satellites over China’s airspace. Using IGS broadcast ephemeris, a simulation was conducted for GPS-visible satellites over China from 00:00 on 29 May 2023, to 00:00 on 30 May 2023. The figure below shows the number of observable GPS satellites over China’s airspace for each hour of the day. The horizontal coordinate represents time in hours within a 24-h day, and the vertical coordinate represents the number of observed satellites at that point. The blue line represents the total number of satellites observed over China’s airspace at a given moment, while the four different colors represent the number of satellites observed at that moment from the four selected points, with red representing the number of satellites observed at the easternmost point where the center line of the main channel of the Heilongjiang and Wusuli River intersects, green representing the number of satellites observed on the Pamir Plateau of the westernmost point in Xinjiang, yellow representing the number of satellites observed at the southernmost point in Zengmu Ansha, and purple representing the number of satellites observed at the northernmost point in Mohe.

**Figure 10:**
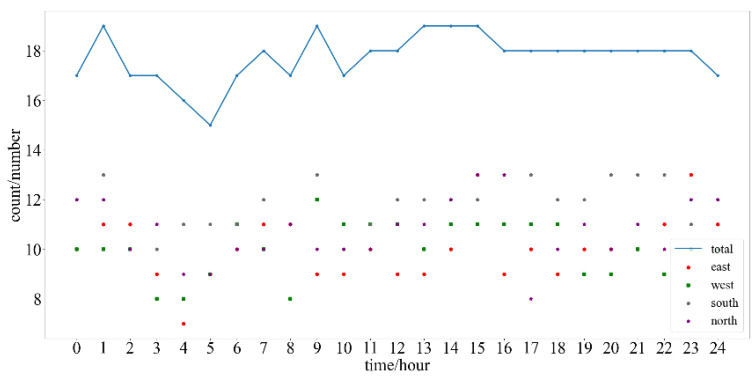
The number of observable GPS satellites over China at each hour from 00:00 on 29 May to 00:00 on 30 May 2023.

From Figure 10, it can be observed that the number of observable GPS satellites over China does not exceed 19 throughout the day. According to the MT2–5 message format, each type of message can broadcast 13 satellites at a time, so it is not necessary to broadcast the full MT2–5, only MT2 and MT3. Therefore, it is feasible to replace MT4 with other types of messages.

(2) The feasibility of decreasing clock error broadcast frequency in MT2–4

**Figure 11:**
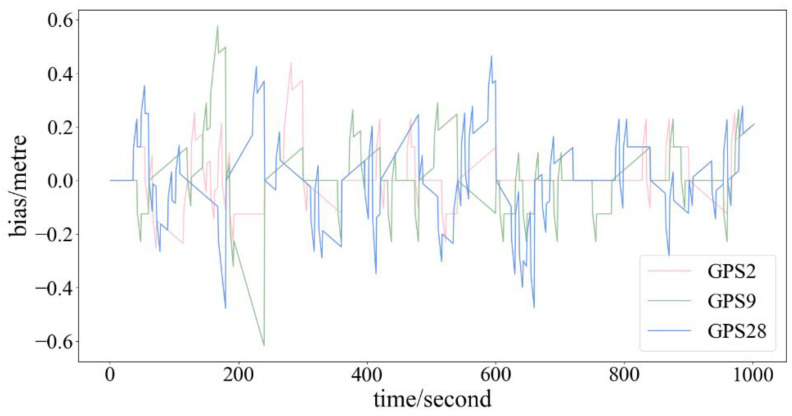
The clock error bias between the intervals of 6 s and 60 s for GPS2.

The real data broadcast by S137 in BDSBAS are processed. The clock error bias between period 6 s and period 60 s for each GPS in the MT2–4 fast correction message over a period of 1000 s is shown in Figure 11, in which the vertical coordinate is the clock error bias (unit: meter) and the horizontal coordinate is the time (unit: second).

Due to errors introduced by receiver satellite clocks and atmospheric propagation, pseudo-range occurs. The corresponding pseudo-range for clock error varies only slightly within 60 s, all remaining below 0.5 m. This indicates that there is minimal impact on positioning due to these clock corrections. Therefore, it is considered feasible to change the interval of fast correction messages from 6 s to 60 s in the third scheduler. In this case, the fixed sequence includes static MT1, MT28, and fast correction messages, without the inclusion of MT24.

According to Figure 11, it can be observed that the clock error bias for three different GPS satellites between the intervals of 6 s and 60 s is less than 0.6m, and the bias is within the acceptable range.

By parsing the actually received messages and considering how the clock difference parameter is distributed among the various parts of the message [23], it is possible to perform the following calculations:

GPS2 in 61–120 s, if the MT2–4 broadcast interval is 6 s, its effective value, i.e., the Root Mean Square (RMS):

(6) $$X_{RMS}=\sqrt{\frac{\sum_{i=60}^{120}{X_{i}}^{2}}{60}}=\sqrt{\frac{0.25^{2}+0.27083^{2}+\ldots +0.35417^{2}}{60}}=0.17349$$

If the MT2–4 broadcast interval is 60 s, its valid value is the intermediate value of the segment’s primary function, i.e.,

(7) $$\frac{0.25+0.37292}{2}=0.31146$$

△ = 0.31146 − 0.17349 = 0.13797(8)

By calculation, it can be found that clock error and clock error parameter bias between the two different broadcast intervals have reached the decimeter level. Therefore, it is feasible to consider decreasing the interval of MT2–4 to 60 s.

### 3.5. Four Fixed Time Sequence Dynamic Message Scheduler

The fixed time sequence dynamic message scheduler refers to broadcasting some messages at fixed intervals while broadcasting the remaining messages in a dynamic time sequence.

When designing the message scheduler, the following specific message types are set in a fixed time sequence:

(1) MT1 uses 210 bits to broadcast PRN masks. The data storage area of 210 bits can store PRN masks corresponding to 210 PRN numbers. A PRN mask of 0 indicates that the satellite corresponding to that PRN number is not monitored by the system, while a PRN mask of 1 indicates that the satellite corresponding to that PRN number is monitored. It has a fixed update interval of 120 s.
(2) MT2–4 are mainly used to broadcast the fast corrections of satellites. The fast corrections and the user differential distance error index (UDREI) in MT2 are the data corresponding to the first 13 satellites with PRN mask 1 in MT1. MT3 includes the data corresponding to satellites 14–26, and MT4 includes the data corresponding to the satellites 27–39. Types 2–4, integrity parameter messages, are broadcast at fixed intervals of 6 s to improve the redundancy of user integrity parameters and meet the system integrity index requirements.
(3) The authentication message MT20 is broadcast at fixed intervals of 6 s and signs the previous five messages.
(4) MT24 mainly broadcasts a mix of satellite fast corrections and long-term corrections. The first half of the message is six fast-change correction data arranged according to the PRN mask sequence, and the second half contains 106 bits of data storage for long-term correction information. It has a fixed update interval of 6 s.
(5) MT28 is primarily used to broadcast covariance matrix information related to satellite orbit and clock correction. Each 250-bit segment per second contains covariance parameters for two satellites, which reduces the effect of errors in positioning. It has a fixed update interval of 120 s.
(6) MT63 is responsible for broadcasting null data and is mainly used to fill broadcasting gaps. Inserting MT63 into the message scheduler allows for bandwidth reservation for new functions, providing redundancy. In the case of Stanford University’s fixed time sequence message scheduler for single frequency L1 SBAS messages, it includes 12 s of MT63 within every 108 s, accounting for 11.1%. Additionally, an analysis of received real messages, including India’s GPS Aided GEO Augmented Navigation (GAGAN) and Japan’s Multi-Functional Satellite Augmentation System (MSAS), shows that in GAGAN, S127 has an MT63 percentage of 25.5%, S132 has 26.1%, and in MSAS, S137 has a 14.3% MT63 usage. Therefore, a certain bandwidth needs to be allocated for MT63 in messages. In this paper, the redundancy is appropriately reduced due to the authentication that has been added to the SBAS message, which is set to broadcast MT63 every 12 s.

With the exception of several messages that are broadcast in a fixed time sequence, the remaining messages are broadcast in a dynamic time sequence. They are inserted into the remaining time slots based on the weight values and priorities of message broadcasting.

This paper introduces authentication into the existing SBAS system, considering SARPs message requirements and SBAS bandwidth optimization principles. It proposes the SBAS message scheduling method with a fixed time sequence message scheduler. Authentication refers to the addition of message types MT20 and MT21. Since MT21 has three functions, this paper treats it as three independent message types: MT21-1, MT21-2, and MT21-3. To determine the optimal bandwidth optimization solution, four schedulers based on the three strategies mentioned in the previous section are presented in Table 4.

### 3.6. Evaluation Metrics

To evaluate the four SBAS message schedulers mentioned above, the evaluation primarily focuses on three aspects: message broadcast intervals, effective message ratio, and TTFF for user receivers.

- Message broadcast intervals

This refers to the time interval between two SBAS messages of the same type received by the user, and it must comply with the maximum update interval specified in the International Civil Aviation Organization (ICAO) SARPs for SBAS messages. Reducing the actual update interval of messages can reduce the impact of correction and integrity parameter delay characteristics on service degradation.

- Effective message ratio

This is the percentage of valid messages other than null messages among all messages. Increasing the effective message ratio means that users receive more useful message information and a decrease in the proportion of MT63 null messages. It indicates an increase in the availability of SBAS messages, thereby improving the integrity requirements for users.

- TTFF

TTFF is the time from the start of the reception of the navigation message to the reception of the data required for a complete position calculation after the user receiver is switched on and has captured the satellite signal [17]. SBAS mainly broadcasts satellite orbit clock correction, ionospheric delay correction and integrity information, which makes the SBAS messages require a large amount of bandwidth. The use of SBAS in systems with short operation times requires minimizing the SBAS TTFF [24].

## 4. Simulation Experiment

### 4.1. Simulation Configuration

The simulation is divided into two steps. The first is to verify the feasibility of adding authentication messages. The next step is to optimize the clock error and reduce the bandwidth occupied by the clock error.

In order to verify the feasibility of adding authentication messages, the authentication message MT20 MT21 is added to the dynamic message schedule designed by Yun Y in Korea. Broadcast static MT1, fast correction messages (MT2, MT3 and MT4 with a broadcast period of 6 s), and authentication message MT20 in a fixed time sequence. The remaining messages are broadcast dynamically. This method is set as the base scheme.

Further, reduce the bandwidth occupied by the clock error in order to mitigate the impact of the authentication message MT20, which occupies 1/6 of the bandwidth, on the SBAS service. There are two approaches: satellite reduction and frequency reduction. In the case of satellite reduction, on the base scheduler, replace MT4 with MT24 in scheduler 1; delete MT4 and change static MT1 to dynamic MT1 in scheduler 2. In the case of frequency reduction, on the base scheduler, reduce the frequency of MT2–4 to 60 s and add MT6 to ensure 6 s integrity in scheduler 3; delete MT4, reduce the frequency of MT2–3 to 60 s, and add MT6 to ensure 6 s integrity in scheduler 4.

Through the above simulation configuration, the feasibility of adding authentication messages is first verified, based on which four message schedulers are proposed and the optimal scheme is selected from them.

### 4.2. Simulation Process

The simulation includes fixed scheduling settings and dynamic scheduling settings.

Fixed scheduling settings mainly broadcast fast messages updated every 6 s, including MT2, MT3, MT24, and MT20, and messages updated every 120 s like MT1 and MT28.

Dynamic scheduling settings involve assigning different weights to message types that are not part of the fixed schedule. The specific policy is as follows:

(1) Prioritize broadcasting messages with the highest current weight value

Set the broadcast weight values for each message type, where the denominator of the weight value is the maximum update interval of the message in the ICAO regulations, and the numerator is the time interval since the last broadcast of that message type. When a new second begins, prioritize broadcasting the message type with the highest current weight value. After broadcasting, set the numerator of the weight value for that message type to 0 [8].

(2) Prioritize broadcasting messages with smaller message priority indices

If multiple message types have the same weight value, select one of the message types for broadcasting based on their message priority index. The message priority index is related to the maximum valid duration of messages defined by the ICAO. The smaller the message priority index, the higher the priority, as shown in Table 5.

During system initialization, it is necessary to read all the message types broadcast by the SBAS, the denominator values of the weighting coefficients for each message type (i.e., the maximum update interval defined in regulations) and the table corresponding to the priorities of each message type, and initialize all the numerator values of the message weights. After initializing the numerator values *A_i_* for each message type, these values are divided by the denominator values *T_i_* to calculate the weights *W_i_*, which will be used for comparison later.

(9) $$W_{i}=\frac{A_{i}}{T_{i}}$$

With each second that passes, the numerator values for all message types increase by 1. Consequently, the weights for all message types involved in dynamic scheduling are updated. By comparing these updated weights, the message type with the highest weight is selected for broadcast. After broadcast, the numerator value for that message type is reset to 0.

(10) $$\max{W_{i}}^{\prime }=\frac{A_{i}+1}{T_{i}}$$

If there are two identical maximum weights, the message with a higher priority, as defined in the message priority table, will be broadcast first. This method also considers message alarms and the situation where there is a change in the PRN number of a satellite.

### 4.3. Simulation Analysis

Simulation experiments include two aspects: first, adding authentication messages to the existing SBAS message scheduler to validate the feasibility of authentication messages; second, validating the four proposed schemes based on optimization strategies by evaluating metrics to select the best one.

(1) Basic scheme

To verify the addition of navigation authentication messages MT20 and MT21 on the basis of WAAS, an evaluation was performed based on the proportion of each message type and the maximum update interval for each message type, which lays the foundation for the subsequent optimization of the scheme after adding authentication. Previously, various schedulers did not include authentication messages. In this paper, the designed schedulers were improved by adding authentication messages and incorporating fixed time sequence messages MT1 and MT63, making more effective use of bandwidth.

SBAS message scheduler includes broadcasting static MT1, fast correction messages (MT2, MT3 and MT4 with a broadcast period of 6 s), and authentication message MT20 using a fixed time sequence. The remaining messages are broadcast dynamically. The specific simulation results are shown in Table 6 and Table 7 below.

Table 6 shows the proportions of various message types. A comparison is made between the scheduler designed in this paper and the results of the dynamic message scheduler designed by Yun Y from South Korea. The WAAS scheme is the simulation result of the dynamic message scheduler of Yun. From the simulation results, it can be observed that the scheduler designed in this paper mainly achieves the inclusion of authentication messages by reducing the bandwidth allocated to dynamic messages MT25, MT26 and MT28, while retaining some redundancy in MT63. The following table is obtained:

**Table 6:** Comparison of the proportions (%) of various message types before and after adding authentication messages to the WAAS messages.

Scheme	MT2	MT3	MT4	MT25	MT26	MT28
WAAS	16.67	16.67	16.67	12.9	8.0	9.8
the scheduler designed in this paper	16.67	16.67	16.67	3.01	1.30	3.01
**Scheme**	**MT63**	**MT20**	**MT21-1**	**MT21-2**	**MT21-3**	**etc.**
WAAS	11.5	-	-	-	-	7.73
the scheduler designed in this paper	8.33	16.67	3.01	1.11	1.11	12.46

The “percentage of each message type” in the table refers to the ratio of the number of times the message is received in the simulation within a certain period of time to the total number of times the simulation is performed. And “etc.” includes MT1, MT7, MT9, MT10, MT17, and MT18.

**Table 7:** Comparison of the maximum update intervals (s) for each message type before and after adding authentication messages to the WAAS messages.

Scheme	MT2	MT3	MT4	MT25	MT26	MT28	MT63	MT20	MT21-1	MT21-2	MT21-3
WAAS	6	6	6	85	288	103	Not mentioned	-	-	-	-
the scheduler designed in this paper	6	6	6	37	89	42	12	6	37	101	103

According to Table 6, the proportions of MT25, MT26, and MT28 have significantly decreased, collectively decreasing by approximately 23%. This bandwidth reduction can be allocated to accommodate authentication messages. With the addition of authentication messages, the proportion of MT63 is controlled at 8.33%, reducing null messages and improving bandwidth utilization while maintaining a certain level of redundancy, thus providing feasibility for adding new functionality. As shown in Table 7, after adding authentication messages, the maximum update intervals for each message type still meet the maximum update intervals stipulated by the ICAO. Dynamic messages such as MT25, MT26, and MT28 exhibit a phenomenon where their proportions decrease while their maximum update intervals decrease as well. This occurs because dynamic messages are not broadcast in a fixed proportion but rather based on weight values and priorities. This can lead to a decrease in proportion and a reduction in the maximum update interval. By efficiently utilizing bandwidth while adhering to the ICAO maximum update interval requirements, the addition of authentication messages to existing messages may be considered.

(2) Comparison and analysis of four fixed time sequence dynamic message schedulers

**Figure 12:**
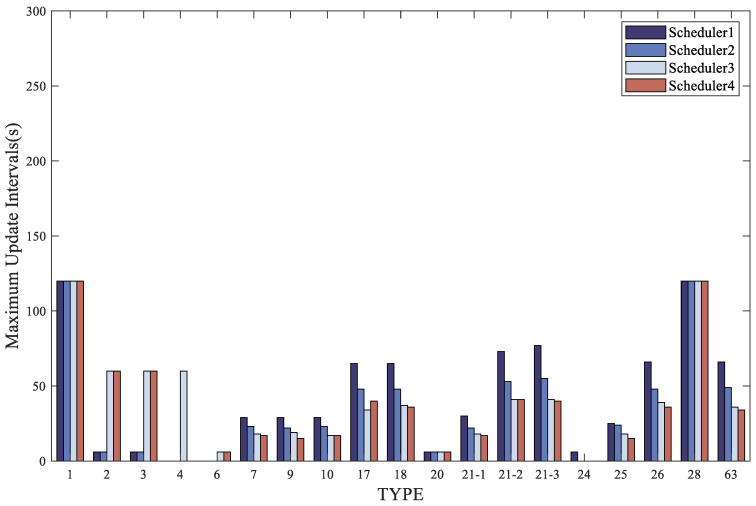
Comparison of the maximum update intervals (s) for the four schedulers after adding authentication.

**Figure 13:**
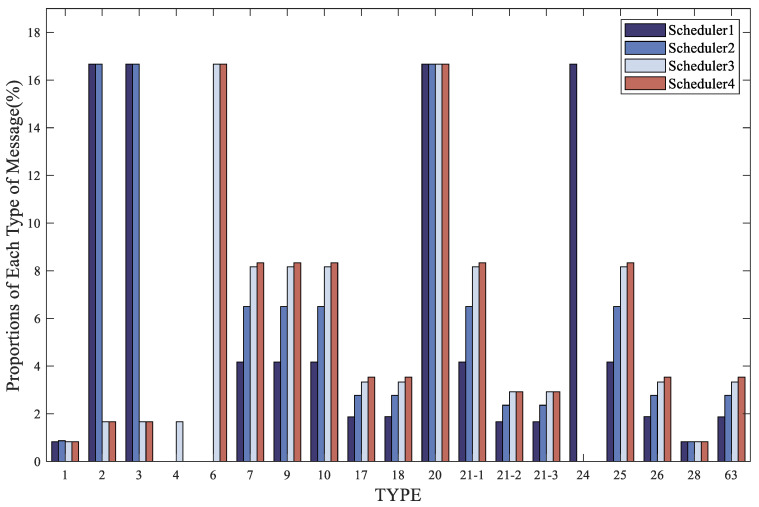
Comparison of the proportions (%) of each type of message for the four schedulers after adding authentication.

Furthermore, a comparison was made among the four optimization schemes to optimize the bandwidth after the addition of authentication messages. The simulation results for the proportion and maximum update interval of each type of message after adding authentication are shown in Figure 12 and Figure 13. It can be observed from the figures that none of the four schedulers exceeded the message broadcast timeout, thus satisfying the maximum update interval specified by the ICAO. The presence of dynamic messages in the schedulers provides available bandwidth for the addition of authentication messages, thus confirming the feasibility of the method proposed in this paper.

The fixed time sequence messages, as their cycles are predefined, are broadcast according to fixed intervals. In the first scheduler, MT24 is used to replace MT4 as a fixed time sequence message. This is because in practical broadcast messages, the fast correction information can be transmitted by MT2 and MT3 together, and MT24 contains mixed correction information, including fast correction information. Since both MT24 and MT4 have a fixed interval of 6 s, their proportions are the same, at 16.67%. In the second scheduler, dynamic MT1 is used. According to the requirements of dynamic MT1, it is set to appear once every 1–2 h, indicating that the status of observed satellites has changed and there is a change in the PRN mask. At this time, MT1 is broadcast repeatedly to ensure that users receive the latest PRN mask. Since MT1 has a fixed interval of 120 s, it is broadcast once every 120 s, with a repeat of MT1 occurring every 3600–7200 s. This repetition of MT1 has a relatively minor impact on other messages during a day of message broadcasting. According to the table, in the second scheduler, since there is no MT4 occupying 1/6 of the bandwidth, the proportion of dynamic messages is higher compared to the first scheduler, and the maximum update intervals of dynamic messages are shorter than that in the first scheme. In the third scheduler, the fast correction message interval is set to 60 s. For integrity requirements, MT6 is introduced for integrity monitoring, broadcast every 6 s, which is the same interval as the fast correction messages MT2–4 in the first two schedulers. MT6 has the same proportion as the fast correction messages MT2–4 in the first two schedulers, at 16.67%. However, for other messages, due to the increased interval of MT2–4, their proportions will inevitably decrease. The reduced proportion is then allocated to other messages. The proportions of other dynamic messages in this scheduler are higher compared to the first two schedulers, and the maximum update interval for these messages is shorter than in the first two schedulers. In the fourth scheduler, MT4 is canceled, and the frequency of MT2–5 is reduced at the same time. Similar to the third scheduler, the fast correction message interval is changed to 60 s. For integrity requirements, MT6 is introduced for integrity monitoring. This scheduler is an improvement over the third scheduler. Since MT4 is canceled, the proportions of various dynamic messages are increased compared to the third scheduler, and the maximum update interval for these messages is reduced compared to the third scheduler. As shown in Table 8 and Table 9, the third and fourth schedulers have shorter maximum update intervals for messages, which satisfies integrity requirements while also allowing for increased proportions of other messages and more information to be broadcast in these messages.

In terms of the initial broadcast times, as shown in Table 10, the first broadcast time for MT20 is manually set in the simulation, occurring at around 3 or 4 s. For the remaining four types of messages, their priority is determined by the value of the denominator in their weight calculations, with values ranging from 21-1, 26, 21-2, to 21-3 (21-2 and 21-3 share the same denominator of 360). A smaller denominator results in a higher weight value ratio. Prioritization follows from high to low: 21-1, 26, 21-2, and 21-3. Consequently, the first appearance times are as follows, from earliest to latest: 21-1, 26, 21-2, and 21-3. In the second scheduler, the repeated broadcast of MT1 has minimal impact on the message broadcast, as the repetition of MT1 occurs only once every 3600–7200 s, which does not significantly affect the initial broadcast times. However, with the elimination of MT4, which occupied 1/6 of the bandwidth, the remaining dynamic messages are broadcast earlier than in the first scheduler. In the third scheduler, due to the increased interval of MT2–4, they do not need to be broadcast every 6 s, and other messages are broadcast ahead of schedule compared to the first two schedulers. The initial positioning time for MT26 is reduced by nearly half compared to the first scheduler, the interval of the fast authentication message MT20 is fixed at 6 s, and the initial authentication time for the slow authentication message MT21, is also reduced by almost half. In the fourth scheduler, MT4 is canceled on the basis of the third scheduler, and the bandwidth saved from MT4 is allocated to other messages. Consequently, the initial broadcast times for various message types are generally improved compared to the third scheduler.

Taking the above into account, based on the effective bandwidth utilization and the shortest TTFF, the fourth scheduler is the most optimal.

Several message schedulers proposed in this paper comply with the ICAO regulations regarding the maximum update intervals for SBAS messages. Table 11 and Table 12 provide a comparison of the four optimization schedulers and the scheduler designed by Yun Y in Korea for broadcasting WAAS.

Compared to the SBAS fixed time sequence message scheduler, all schedulers show improvements in message update intervals, with the optimal fourth scheduler having a user’s TTFF of only 26 s, compared to the specified maximum update interval of 300 s. This indicates an enhancement in system integrity. Additionally, compared to a fully dynamic message scheduler, the fixed time sequence dynamic message schedulers proposed in this paper can significantly reduce the probability of simultaneous timeout of multiple messages due to the setting of several messages that do not need to participate in the dynamic selection of message broadcasts. Once there is a prolonged system failure or system downtime in the SBAS, this proposed scheduler also leads to faster recovery times compared to a fully dynamic message scheduler [8]. When a message alarm occurs, the SBAS will broadcast an alarm message to the user, causing delays in the transmission of all message types. Setting MT63 null message at a fixed interval of every 12 s, this ratio is lower compared to WAAS, since the fact that authentication has already been added to the message and the remaining redundancy is sufficient to support the addition of new features.

## 5. Conclusions

SBAS is widely used in civil aviation, but there is a risk that it is vulnerable to spoofing attacks. The International Civil Aviation Organization (ICAO) has continued to promote SBAS authentication services to enhance the security of the system. For the problem that SBAS messages need to occupy 1/6 of the bandwidth after joining the authentication, which leads to the shortage of the original bandwidth, this paper proposes an optimized message scheduler based on SBAS clock error messages. Reduces the bandwidth occupied by clock error messages by means of star reduction and frequency reduction. Simulation experiments show that the effective message ratio in SBAS is over 91% for all four schemes, of which the optimal scheme is the fourth one, with the initial user positioning time of 26 s. The optimization schemes proposed in this paper meet the demand of broadcasting SBAS authentication information while retaining a certain amount of bandwidth margin, which provides space for future system upgrades or the addition of new functions.

## Figures and Tables

**Table 1:** Newly defined message content update interval.

Data Type	MT	Maximum Update Interval (s)	Timeout Interval (s)
SBAS L1 Authentication message	20	6 s	-
Root key of the TESLA key chain and signature	21-1	120 s	Identified in the OTAR message
Level 2 public key and signature	21-2	360 s	Identified in the OTAR message
CA public key	21-3	360 s	Identified in the OTAR message

**Table 2:** Single-frequency SBAS message content and maximum update period (RTCA 2006) [20].

MT	Content	Maximum Update Interval (s)
0	“Do Not Use” (SBAS test mode)	6
1	PRN mask	120
2–5	Fast corrections	6
6	Integrity Information	6
7	Fast Correction Degradation Factor	120
9	GEO ranging function parameters	120
10	Degradation Factors	120
12	SBAS network time/UTC offset parameters	300
17	GEO satellite almanacs	300
18	Ionospheric grid point masks	300
20	SBAS L1 Authentication Message	6
21	SBAS Over the Air Rekeying (OTAR)	120/360
24	Mixed fast/long-term satellite error corrections	6
25	Long-term satellite error corrections	120
26	Ionospheric delay corrections	300
27	SBAS service message	300
28	Clock-ephemeris covariance matrix	120
62	Reserved	
63	Null message	300

**Table 3:** PRN mask assignments [21].

PRN Slot	Assignment
entry 1	data
1–37	GPS/GPS Reserved PRN
38–61	GLONASS Slot Number plus 37
62–119	Future GNSS
120–158	GEO/SBAS PRN
159–210	Future GNSS

**Table 4:** Four schedulers after adding authentication.

Scheduler	Messages Broadcast in a Fixed Time Sequence	Remaining Messages (Include MT21)
Scheduler 1	Static MT1 + MT28 + MT63 + Fast corrections (MT2, MT3) (6s) + Authentication message MT20 + MT24	7 9 10 17 18 26
Scheduler 2	dynamic MT1 + MT28 + MT63 + Fast corrections (MT2, MT3) (6s) + Authentication message MT20(Notes: excluding MT24)	7 9 10 17 18 25 26
Scheduler 3	Static MT1 + MT28 + MT63 + Fast corrections (MT2, MT3, MT4) (60s) + Authentication message MT20 + MT6 (6s) (Notes: excluding MT24)	7 9 10 17 18 25 26
Scheduler 4	StaticMT1 + MT28 + MT63 + Fast corrections (MT2, MT3) (60s) + Authentication message MT20 + MT6 (6s) (Notes: excluding MT24)	7 9 10 17 18 25 26

**Table 5:** Message priority index.

MT	Message Priority Index
25	1
28	2
7	3
10	4
9	5
21-1	6
18	7
26	8
17	9
63	10
21-2	11
21-3	12

**Table 8:** Comparison of the maximum update intervals (s) for various message types in the four schedulers after adding authentication.

Scheduler	MT1	MT2	MT3	MT4	MT6	MT7	MT9	MT10	MT17	MT18
1	120	6	6	-	-	36	36	36	90	90
2	120	6	6	-	-	24	24	28	53	50
3	120	60	60	60	6	22	21	22	42	36
4	120	60	60	-	6	21	22	21	40	40
**Scheduler**	**MT20**	**MT21-1**	**MT21-2**	**MT21-3**	**MT24**	**MT25**	**MT26**	**MT28**	**MT63**	
1	6	36	97	107	6	36	90	120	12	
2	6	24	65	65	-	24	53	120	12	
3	6	21	48	42	-	21	45	120	12	
4	6	21	49	44	-	21	40	120	12	

**Table 9:** Comparison of the proportions (%) of various message types in the four schedulers after adding authentication.

Scheduler	MT1	MT2	MT3	MT4	MT6	MT7	MT9	MT10	MT17	MT18
1	0.83	16.67	16.67	-	-	3.33	3.33	3.33	1.39	1.39
2	0.87	16.67	16.67	-	-	5.66	5.66	5.66	2.50	2.50
3	0.83	1.67	1.67	1.67	16.67	7.33	7.33	7.33	3.33	3.33
4	0.83	1.67	1.67	-	16.67	7.61	7.61	7.61	3.24	3.24
**Scheduler**	**MT20**	**MT21-1**	**MT21-2**	**MT21-3**	**MT24**	**MT25**	**MT26**	**MT28**	**MT63**	
1	16.67	3.33	1.25	1.25	16.67	3.33	1.39	0.83	8.33	
2	16.66	5.66	2.08	2.08	-	5.66	2.50	0.83	8.33	
3	16.67	7.33	2.50	2.50	-	7.33	3.33	0.83	8.33	
4	16.67	7.61	2.78	2.78	-	7.61	3.24	0.83	8.33	

**Table 10:** Comparison of initial broadcast times (s).

Scheduler	MT20	MT21-1	MT21-2	MT21-3	MT26
1	4	29	89	96	65
2	3	17	53	54	36
3	4	15	38	42	27
4	3	14	38	41	26

**Table 11:** Comparison of the maximum update intervals (s) for the four optimization schedulers and the WAAS scheduler.

Scheme	MT2	MT3	MT4	MT25	MT26	MT28	MT20	MT21-1	MT21-2	MT21-3
WAAS	6	6	6	85	288	103	-	-	-	-
Scheduler 1	6	6	-	36	90	120	6	36	97	107
Scheduler 2	6	6	-	24	53	120	6	24	65	65
Scheduler 3	60	60	60	21	45	120	6	21	48	42
Scheduler 4	60	60	-	21	40	120	6	21	49	44

**Table 12:** Comparison of the proportions (%) of each type of message between the four optimization schedulers and the WAAS scheduler.

Scheme	MT2	MT3	MT4	MT25	MT26	MT28
WAAS	16.67	16.67	16.67	12.9	8.0	9.8
Scheduler 1	16.67	16.67	-	3.33	1.39	0.83
Scheduler 2	16.67	16.67	-	5.66	2.50	0.83
Scheduler 3	1.67	1.67	1.67	7.33	3.33	0.83
Scheduler 4	1.67	1.67	-	7.61	3.24	0.83
**Scheme**	**MT63**	**MT20**	**MT21-1**	**MT21-2**	**MT21-3**	**MT6**
WAAS	11.5	-	-	-	-	-
Scheduler 1	8.33	16.67	3.33	1.25	1.25	-
Scheduler 2	8.33	16.67	5.66	2.08	2.08	-
Scheduler 3	8.33	16.67	7.33	2.50	2.50	16.67
Scheduler 4	8.33	16.67	7.61	2.78	2.78	16.67

## Data Availability

The IGS broadcast ephemeris can be found in the IGS Data Center at www.igs.gnsswhu.cn/ (accessed on 29 May 2023).
